# Prevention of healthcare-associated infections: ethical perspectives and professional responsibilities

**DOI:** 10.25122/jml-2026-0030

**Published:** 2026-05

**Authors:** Carmen Mihaela Ioniță

**Affiliations:** 1 Carol Davila University of Medicine and Pharmacy, Bucharest, Romania

**Keywords:** healthcare-associated infections, infection prevention, patient safety, professional responsibility, medical ethics, bioethics

## Abstract

Healthcare-associated infections represent a major public health problem, significantly impacting patient safety and the quality of medical care. Beyond their clinical and epidemiological implications, the prevention of these infections raises important ethical issues. Healthcare professionals have both a professional and moral responsibility to adopt all necessary measures to reduce the risk of healthcare-associated infections. The present article aimed to analyze the ethical dimension of preventing healthcare-associated infections, highlighting the role of fundamental bioethical principles, such as beneficence, non-maleficence, and professional responsibility, in guiding clinical practice. In addition, the responsibilities of healthcare personnel and the ethical dilemmas that may arise in preventing and reporting these infections are discussed. The integration of bioethical principles into medical practice improves the quality of healthcare and strengthens the relationship of trust between patients and healthcare professionals.

## Introduction

Healthcare-associated infections are among the most important public health challenges worldwide, significantly impacting patient safety and the quality of medical care [1-3]. These infections are frequently associated with prolonged hospital stays, increased healthcare costs, and, in some cases, severe complications that may endanger the patient’s life [3,4].

According to data published by the World Health Organization (WHO), hundreds of millions of patients worldwide are affected each year by healthcare-associated infections [1]. Studies indicate that approximately 7% of hospitalized patients in developed countries and 10–15% in developing countries may develop such infections during hospitalization [2,5]. These infections are among the most frequent complications of medical care and pose a major challenge for healthcare systems from both clinical and economic perspectives [3,6].

In Romania, healthcare-associated infections pose a significant challenge to the healthcare system. National data indicate a gradual increase in the reporting of these infections in recent years, as a result of the implementation of surveillance and control programs for nosocomial infections [7,8]. The monitoring of these infections is coordinated by institutions such as the National Institute of Public Health, which collects and analyzes data regarding the incidence of healthcare-associated infections [7]. Increasing awareness and implementing prevention measures contribute to improving patient safety and developing responsible medical practices [9].

Beyond their clinical and epidemiological dimensions, healthcare-associated infections also raise a number of ethical concerns [10]. Healthcare professionals have both a professional and a moral responsibility to adopt all necessary measures to prevent such complications, in accordance with the fundamental principles of bioethics, such as beneficence, non-maleficence, and respect for the patient [11,12].

In contemporary medical practice, the prevention of healthcare-associated infections is not only a professional obligation but also an ethical responsibility of the entire healthcare team [10,13]. Adherence to prevention protocols, proper application of hygiene measures, and careful patient monitoring are essential to protecting patient health and safety.

The aim of this article was to analyze the ethical responsibility of healthcare professionals in preventing healthcare-associated infections, highlighting the relationship between bioethical principles and clinical practices to reduce the risk of these complications.

## Material and Methods

This study was designed as a narrative review based on a comprehensive analysis of the existing literature on healthcare-associated infections and their ethical implications. Relevant sources were identified through major databases, including PubMed, as well as reports and guidelines from international organizations such as the World Health Organization (WHO), the Centers for Disease Control and Prevention (CDC), and the European Center for Disease Prevention and Control (ECDC).

The selection criteria included publications addressing infection prevention, patient safety, and ethical responsibilities in clinical practice. Priority was given to recent studies, international guidelines, and foundational works in bioethics.

The aim of this methodological approach was to integrate key bioethical principles—beneficence, non-maleficence, autonomy, and justice—into the framework of healthcare-associated infection prevention, providing a structured ethical perspective on clinical practice.

### The ethical dimension of preventing healthcare-associated infections

The prevention of healthcare-associated infections is not only a clinical or epidemiological issue, but also involves an important ethical dimension [1,3,6]. In medical practice, every intervention or act of care implies the assumption of responsibility toward the patient, a responsibility derived from the fundamental principles of bioethics [4].

One of the central principles of bioethics is non-maleficence, which involves avoiding harm to the patient. In the context of preventing healthcare-associated infections, this principle requires adopting all necessary measures to reduce the risk of contamination and protect patients from avoidable complications [5,10]. Failure to comply with preventive measures, such as proper hand hygiene or the use of sterile materials, may lead to infections that could otherwise have been prevented [2,7,12].

In addition, the principle of beneficence involves acting in the patient’s best interests and promoting their health and well-being. In this regard, healthcare professionals have a moral obligation to apply the best available practices for infection prevention, thereby improving the quality of medical care and enhancing patient safety [1,9].

Respecting these ethical principles helps strengthen the relationship of trust between patients and healthcare professionals. Patients expect medical care to be provided under conditions of maximum safety, and the prevention of healthcare-associated infections represents an essential element in maintaining this trust [1].

### The responsibility of healthcare professionals in preventing healthcare-associated infections

The prevention of healthcare-associated infections represents a fundamental responsibility of the entire healthcare team. In medical practice, every healthcare professional contributes to maintaining a safe environment for patients by adhering to prevention protocols and applying care procedures correctly [6,13].

Healthcare professionals have both a professional and a moral obligation to adhere to hygiene standards and contamination prevention measures, such as rigorous hand hygiene, the use of protective equipment, and the proper application of aseptic and antiseptic techniques. These measures are essential for reducing the risk of microorganism transmission and preventing complications associated with medical care [7,12].

From an ethical perspective, adherence to these protocols reflects healthcare professionals’ commitment to the fundamental principles of bioethics, particularly non-maleficence and beneficence [4]. By properly applying preventive measures, healthcare professionals help protect patients from avoidable risks and ensure high-quality medical care [1].

At the same time, the ethical responsibility of healthcare professionals also involves maintaining a constant level of professional competence. Participation in continuing education programs and the ongoing updating of knowledge on infection prevention are essential for adapting to new recommendations and improving clinical practices [5,10].

### The impact of healthcare-associated infections on patients

Healthcare-associated infections represent a major challenge for healthcare systems worldwide, having significant consequences for both patients and medical institutions [1,6]. The occurrence of these infections may lead to important medical complications, prolonged hospitalization, and an increased risk of morbidity and mortality [11,14].

Beyond their clinical impact, these infections may also significantly affect patients’ psychological well-being. The development of an infectious complication during hospitalization may generate anxiety, mistrust, and a sense of vulnerability. Patients may perceive such situations as a consequence of deficiencies within the healthcare system, which can negatively affect the relationship of trust between patients and healthcare professionals [14].

In addition, healthcare-associated infections frequently generate additional costs for healthcare systems due to the need for supplementary treatments, further diagnostic investigations, and extended hospital stays [11]. These aspects highlight the importance of implementing effective strategies for infection prevention and control [5].

From an ethical perspective, the prevention of healthcare-associated infections represents an essential component of respecting patients’ rights and the fundamental principles of bioethics [4]. Protecting patients from avoidable risks should be a priority for any healthcare system and for every professional involved in medical care [1].

### Ethical dilemmas in the prevention of healthcare-associated infections

The prevention of healthcare-associated infections involves not only compliance with clinical protocols but also the management of complex ethical dilemmas that may arise in medical practice. In certain situations, healthcare professionals face difficult decisions regarding professional responsibility, patient transparency, and the accurate reporting of medical incidents [6].

One frequently discussed ethical dilemma concerns the reporting of healthcare-associated infections. In some cases, there may be reluctance to report such situations due to concerns about the institution’s reputation or potential administrative consequences. From an ethical perspective, transparency and the accurate reporting of these events are essential for improving medical practices and protecting patients [1,3].

Another important ethical dilemma relates to the balance between individual and institutional responsibility in preventing infections. Although each member of the healthcare team is responsible for complying with prevention protocols, medical institutions are also obligated to ensure the necessary conditions for the proper implementation of these measures, including adequate resources and professional training programs [5,6].

At the same time, maintaining open and honest communication with patients represents an essential component of ethical medical practice. Informing patients about the risks associated with medical care and any complications that may arise during hospitalization helps strengthen the relationship of trust between patients and healthcare professionals [9].

The analysis of these dilemmas highlights the fact that the prevention of healthcare-associated infections should be approached not only as a technical or organizational issue but also as a moral responsibility shared by all professionals involved in patient care [4].

### Proposed ethical framework for preventing healthcare-associated infections

Based on the analysis of the literature and the ethical principles discussed, this paper proposes a practical framework to guide healthcare professionals in preventing healthcare-associated infections.

This framework includes several key components:

1. Strict adherence to infection prevention and control protocols [5,10];
2. Continuous professional education and training in infection prevention [2,5,12];
3. Ethical responsibility in the accurate reporting of healthcare-associated infections [6,11,13];
4. Transparent and honest communication with patients regarding risks and complications [4,8];
5. Institutional responsibility in ensuring adequate resources, staffing, and safe working conditions [1,5,6].

This structured approach integrates ethical principles into daily clinical decision-making and supports the development of a culture of safety, accountability, and professional responsibility within healthcare systems.

## Conclusion

Healthcare-associated infections continue to represent a persistent challenge that extends beyond clinical management into the realm of ethical responsibility. This paper has emphasized that effective prevention is not limited to adherence to protocols but requires a deeper integration of ethical principles into everyday medical practice.

The analysis highlights that professional responsibility plays a central role in bridging the gap between knowledge and action. While guidelines provide the necessary framework, it is the ethical commitment of healthcare professionals that ultimately determines their consistent application. In this context, ethical dilemmas—such as balancing workload, resource limitations, and patient safety—require not only technical competence, but also critical reflection and moral judgment.

Therefore, strengthening ethical awareness should be considered an essential component of infection prevention strategies. Encouraging accountability, fostering a culture of safety, and promoting continuous ethical engagement can significantly improve compliance and patient outcomes.

In conclusion, a sustainable reduction in healthcare-associated infections depends on aligning clinical practice with ethical responsibility, thereby reinforcing both the quality of care and trust in the healthcare system.
